# The Postnatal Lung Maturation Disrupted by Increased Pulmonary Blood Flow and Its Clinical Implications

**DOI:** 10.1016/j.jacasi.2026.02.013

**Published:** 2026-03-26

**Authors:** Sixie Zheng, Zheng Wang, Yiting Xue, He Zhang, Yingying Xiao, Yuqing Hu, Debao Li, Qing Cui, Chenxi Liu, Jing Wang, Lincai Ye, Lisheng Qiu

**Affiliations:** a Department of Thoracic and Cardiovascular Surgery, Shanghai Children’s Medical Center, Shanghai Jiao Tong University School of Medicine, Shanghai, China; b Department of Pediatrics, The Women and Children's Hospital of Ningbo University, Ningbo, Zhejiang, China; c Department of Thoracic and Cardiovascular Surgery, Shanghai Children’s Hospital, Shanghai Jiao Tong University School of Medicine, Shanghai, China; d Department of Cardiology, Shanghai Children’s Medical Center, Shanghai Jiao Tong University School of Medicine, Shanghai, China; e Department of Pediatric Surgery, Children’s Hospital of Fudan University, National Children’s Medical Center, Shanghai, China; f Department of Infectious Diseases, Shanghai Children’s Medical Center, Shanghai Jiao Tong University School of Medicine, Shanghai, China; g Shanghai Institute for Pediatric Congenital Heart Disease, Shanghai Children’s Medical Center, Shanghai Jiao Tong University School of Medicine, Shanghai, China

**Keywords:** increased pulmonary blood flow, left-to-right shunt, lung, neonatal animal models, pediatric

## Abstract

**Background:**

Increased pulmonary blood flow (IPF) from congenital heart diseases causes pediatric pulmonary hypertension and respiratory distress, yet its impact on postnatal lung maturation remains unknown.

**Objectives:**

This study aimed to establish a neonatal model of IPF and elucidate the molecular mechanisms underlying impaired lung maturation, thereby identifying potential therapeutic targets.

**Methods:**

Neonatal mice underwent surgical creation of an aortocaval fistula to induce IPF. Bulk RNA sequencing compared lung transcriptomes at postnatal day (P)14 (alveolar stage) and P30 (maturity) in IPF vs sham-operated controls. Histological validation was performed, and the effects of immunosuppression (cyclosporine A) were assessed.

**Results:**

IPF generated 2,272 differentially expressed genes vs 943 in controls, revealing the following: 1) shared downregulation of extracellular matrix organization and cell cycle pathways; 2) IPF-specific cell cycle dysregulation (downregulated Birc5/CENPE) and hyperactive immunity (upregulated NLRP3/IL23R) impairing alveolar/capillary development; 3) suppressed circadian (Per2) and neural pathways (Nr1d1) unique to normal maturation. Cyclosporine A treatment mitigated alveolar simplification, attenuated vascular remodeling, and improved alveolar epithelial differentiation.

**Conclusions:**

This study establishes the first neonatal IPF model and identifies actionable therapeutic targets, including immune modulators (NLRP3/IL23R inhibitors), cell cycle regulators (Birc5/CENPE agonists), and neural/circadian mediators (Nr1d1/Per2 activators). These findings provide a roadmap for mitigating IPF-driven lung maldevelopment.

Increased pulmonary blood flow (IPF) refers to a greater than normal volume of blood passing through the pulmonary circulation, which includes the pulmonary arteries, capillaries, and veins. This condition can occur due to various pathological reasons, such as left-to-right shunts, high cardiac output states, pulmonary arteriovenous malformations, and chronic lung diseases.^1^, ^2^, ^3^, ^4^, ^5^ Over time, IPF has significant implications for cardiovascular and respiratory function, such as pulmonary hypertension (PH), heart failure, and cyanosis.^1^, ^2^, ^3^, ^4^, ^5^ In children, IPF is more prevalent than in adults, often due to congenital heart defects such as atrial septal defect, ventricular septal defect, or patent ductus arteriosus,^6^, ^7^, ^8^, ^9^, ^10^, ^11^ as well as pulmonary arteriovenous malformations, including primary or secondary pulmonary vein stenosis and pulmonary vein ectopic drainage.^12^ Approximately 70% of pediatric PH cases are caused by IPF.^13^, ^14^, ^15^ The initial step in treating pediatric PH is addressing the underlying condition, such as surgical repair of congenital defects.^13^, ^14^, ^15^ However, in some cases, the primary disease is difficult to cure, as with pulmonary vein stenosis,^12^^,^^16^ and even after correction, PH may persist.^15^^,^^17^ Importantly, most pediatric PH cases lack effective treatments, and adult PH medications often fail to improve outcomes in pediatric patients.^14^^,^^18^ This may be because of the immaturity of children's lungs, which are still in a critical period of dynamic development, with certain key cells and molecules appearing transiently during this stage and playing a crucial role in regulating lung maturation.^19^^,^^20^ Therefore, investigating how IPF regulates postnatal lung maturation at the molecular levels can not only enhance our understanding of lung development but also provide potential therapeutic targets for pediatric PH.

Postnatal lung maturation can be categorized into 3 interrelated aspects:^21^^,^^22^ 1) alveolar maturation, during which more than 90% of alveoli in mice form between postnatal day (P)1 and P30 (Figure 1A); 2) microcirculation maturation, in which the double capillary networks in the parenchymal septa at P1 are restructured into a single capillary system by P30 to optimize gas exchange (Figure 1B); and 3) neural regulation maturation, which remains poorly understood. Transcriptomic analysis of 3 common inbred mouse strains revealed that postnatal lung maturation can be divided into 5 stages: alveolar development stage 1 (ALV1, P0-P3), ALV2 (P4-P7), ALV3 (P9-P12), ALV4 (P13-P18), and mature lung (P19-P56) (Figure 1C).^23^^,^^24^ To investigate whether and how IPF reshapes postnatal lung maturation, we established a neonatal mouse model at P7 by creating a fistula between the inferior vena cava (IVC) and the abdominal aorta (AA) (aortocaval fistula [ACF]), inducing a left-to-right shunt, as described in previous studies.^25^, ^26^, ^27^, ^28^ Lungs from P14 (ALV4 stage) and P30 (mature stage) were then subjected to bulk RNA sequencing (RNA-seq) analysis and subsequent validation to assess the impact of IPF on postnatal lung maturation.

**Figure 1:**
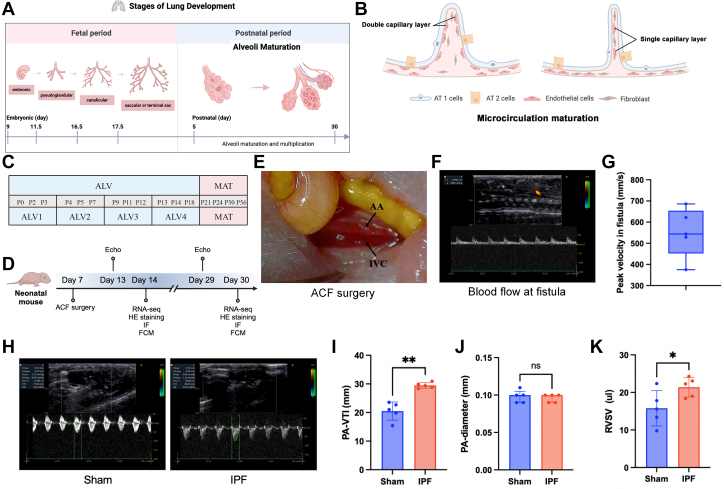
Neonatal ACF Microsurgery Induces IPF (A) Stage of lung development in mice. Noted that from postnatal day (P)5 to P30, there were alveoli maturation, characterized by alveoli multiplication. (B) Microcirculation maturation characterized by the increase of single capillary network. AT1 = Type 1 alveolar cells; AT 2 = Type 2 alveolar cells. (C) Stages of postnatal alveolar development. (D) Study flowchart. (E) Local location of the AA and IVC used for creating the fistula. (F) A representative image of blood flow through the fistula. (G) Quantification of peak velocity at the fistula. n = 5 mice. (H) Representative image of echocardiography at transpulmonary valve. (I) Quantification of pulmonary artery velocity time integral (PA-VTI) in sham and IPF group. n = 5 mice. (J) Quantification of pulmonary artery diameter in sham and IPF group. n = 5 mice. (K) Quantification of right ventricular stroke volume (RVSV) in sham and IPF group. n = 5 mice. ∗*P* < 0.05, ∗∗*P* < 0.01, and ns = not significant. AA = abdominal aorta; ACF = aortocaval fistula; FCM = flow cytometry; HE = hematoxylin-eosin; IF = immunofluorescence; IPF = increased pulmonary blood flow; IVC = inferior vena cava; RNA-seq = RNA sequencing.

## Materials and Methods

### Data availability and ethics statement

The data generated from this study are available from the corresponding author on reasonable request. All RNA-seq data have been deposited in the Gene Expression Omnibus database^29^ with accession numbers GSE280388 and GSE291221. All study procedures complied with the ARRIVE (Animal Research: Reporting of In Vivo Experiments) guidelines and were approved by the Animal Welfare and Human Studies Committee at Shanghai Children's Medical Center (institutional review board approval no. SCMC-LAWEC-2024-1127).

### Study design

As illustrated in Figure 1D, at P7, 60 neonatal pups were randomly assigned to either the sham or IPF group (30 pups per group) and underwent sham or ACF surgery. At P13, all pups underwent abdominal ultrasound and echocardiography to confirm the successful creation of the fistula and the presence of IPF. At P14, 30 pups (15 per group) were subjected to bulk RNA-seq, hematoxylin-eosin (H&E) staining, immunofluorescence staining, and flow cytometry, with 5 pups per experiment. At P29, the remaining 30 pups underwent abdominal ultrasound and echocardiography to further confirm the persistence of the fistula and IPF. At P30, 30 pups (15 per group) were subjected to bulk RNA-seq, H&E staining, immunofluorescence staining, and flow cytometry, with 5 pups per experiment.

### Animals and ACF surgery

C57/BL6 neonatal pups, both male and female, were raised in our laboratory. The pups underwent either ACF surgery or a sham operation, as described in previous publications.^25^, ^26^, ^27^, ^28^ Briefly, after anesthesia with 4% isoflurane, the pups underwent midline laparotomy to expose the AA and IVC. A 9-0 needle (0.07-mm diameter) was used to puncture through the AA into the IVC to create a fistula, generating a left-to-right shunt. After 2 minutes of hemostatic compression, the abdominal wall was closed, and pain relief was provided using local lidocaine treatment. The sham group underwent the same procedure except for the puncture step.

### Abdominal ultrasound and echocardiography

Blood flow at the ACF and pulmonary artery (PA) was analyzed using a Vevo 2100 imaging system (Visual Sonics) as described in previous publications.^25^, ^26^, ^27^, ^28^ The following parameters were measured: velocity time integral (PA-VTI), diameter of the PA valve (D), pulmonary arterial acceleration time, and right ventricle (RV) ejection time. The right ventricular stroke volume (RVSV, mL) and RV systolic pressure (mm Hg) were calculated using the following formulas^30^:

$$\text{RVSV}\;\left[\text{mL}\right]=1/4\times \pi D^{2}\times \text{PA}-\text{VTI}$$

### H&E staining

Lung H&E staining was performed using an H&E staining kit (Solarbio) according to the manufacturer’s protocol. The degree of alveolarization was quantified by measuring the mean linear intercept.^31^^,^^32^

### Immunofluorescence

Paraffin-embedded lung tissues were sectioned into 6-μm slices, dewaxed, hydrated, and subjected to antigen retrieval. After washing 3 times with phosphate-buffered saline (PBS), the slides were permeabilized with 0.5% Triton X-100, blocked with 10% donkey serum at room temperature, and incubated with primary rabbit anti-CD31 antibody at 4°C overnight. The next day, the slides were washed with PBST (PBS with 1% Tween) and incubated with fluorescent secondary antibodies and DAPI (4′,6-diamidino-2-phenylindole) for 1 hour at room temperature.

### RNA-seq

After euthanasia, lung tissues were collected for RNA-seq. Transcriptome sequencing and analysis were conducted by Genechem Biotech Co., Ltd. Total RNA was extracted using the PureLink RNA Micro Scale Kit. RNA quantification and purity were measured using a NanoDrop 2000 spectrophotometer (Thermo Scientific). RNA integrity was assessed with an Agilent 2100 Bioanalyzer (Agilent Technologies). Libraries were constructed using the VAHTS Universal V6 RNA-seq Library Prep Kit according to the manufacturer’s protocol and sequenced on an Illumina NovaSeq 6000 platform to generate 150-base pair paired-end reads. Raw reads in Fastq format were processed using fastp,^33^ and low-quality reads were removed to obtain clean reads. Clean reads were mapped to the reference genome using HISAT.^34^ The fragments per kilobase of transcript per million mapped reads^35^ value of each gene was determined, and gene read counts were calculated using HTSeq-count. Principal component analysis was performed using R (v 3.2.0) to assess biological replication. Differentially expressed genes (DEGs) were identified using the DESeq2 R package (1.16.1). DEGs were defined as those with false discovery rate–adjusted *P* < 0.05 and log2 (fold change) ≥1 using DESeq2.^2^ Gene Ontology (GO) enrichment analysis of DEGs was conducted using the clusterProfiler R package.^36^ Kyoto Encyclopedia of Genes and Genomes (KEGG) pathway enrichment was performed using the clusterProfiler package in R (v.3.18.1), based on the KEGG database.

### Flow cytometry

Lung tissues were minced into small fragments, dissociated with type II collagenase (1,000 U/mL in PBS; Worthington) at 37°C for 30 minutes, and filtered through a 70-μm cell strainer to obtain a single-cell suspension. Red blood cells were removed using red blood cell lysis buffer (eBiosciences). The single-cell suspensions were stained with fluorochrome-conjugated antibodies against CD4 and CD8 (eBiosciences) at 4°C for 30 minutes. Cells were analyzed using a flow cytometer (BD, FACSAriaTM Fusion), and data were analyzed using FlowJo software.

### Quantitative reverse transcriptase polymerase chain reaction

Total RNA was extracted using PureLink RNA Micro Scale Kit. Quantitative polymerase chain reaction was performed using SYBR Green Master Mix according to the manufacturer’s instructions. Relative expression was calculated using the ΔΔCt method and normalized to GAPDH. Primers used are listed in Supplemental Table 1.

### Cyclosporine A intervention

Cyclosporine A (CsA) was injected (12.5 mg/kg/d intraperitoneally, P7-P14) to inhibit immune cell activation during the critical alveolarization window. At P21, the lungs were subjected to immunofluorescence and H&E staining.

### Statistical analysis

Quantitative data are presented as mean ± SD if normally distributed, or as median with IQR if not. The normality of continuous variables was assessed using the Shapiro–Wilk test. Differences between groups were evaluated using Student’s *t*-test for normally distributed data or the Wilcoxon rank-sum test for non-normally distributed data. For experiments involving more than 2 group comparisons, 1-way analysis of variance followed by post hoc pairwise tests was performed. To control the type I error in multiple comparisons, the *P* values were adjusted using the Bonferroni method. Categorical variables were compared using the chi-square test or Fisher’s exact test, as appropriate. A *P* value <0.05 was considered statistically significant. All statistical analyses were performed using SAS software (version 9.4; SAS Institute Inc).

## Results

### Neonatal ACF microsurgery generates IPF

As shown in Figure 1E, the neonatal AA and IVC are located in close proximity. Puncturing through the AA into the IVC created a fistula between the AA and IVC, inducing a left-to-right shunt (Figure 1E). At the fistula site, pulsatile blood flow was observed (Figures 1F and 1G), consistent with previous publications.^25^, ^26^, ^27^, ^28^ In addition, the ACF surgery resulted in increased PA velocity, PA-VTI, and RVSV (Figures 1H to 1K). These findings indicate that the ACF successfully generated IPF.

### Postnatal lung maturation track is altered by IPF

**Figure 2:**
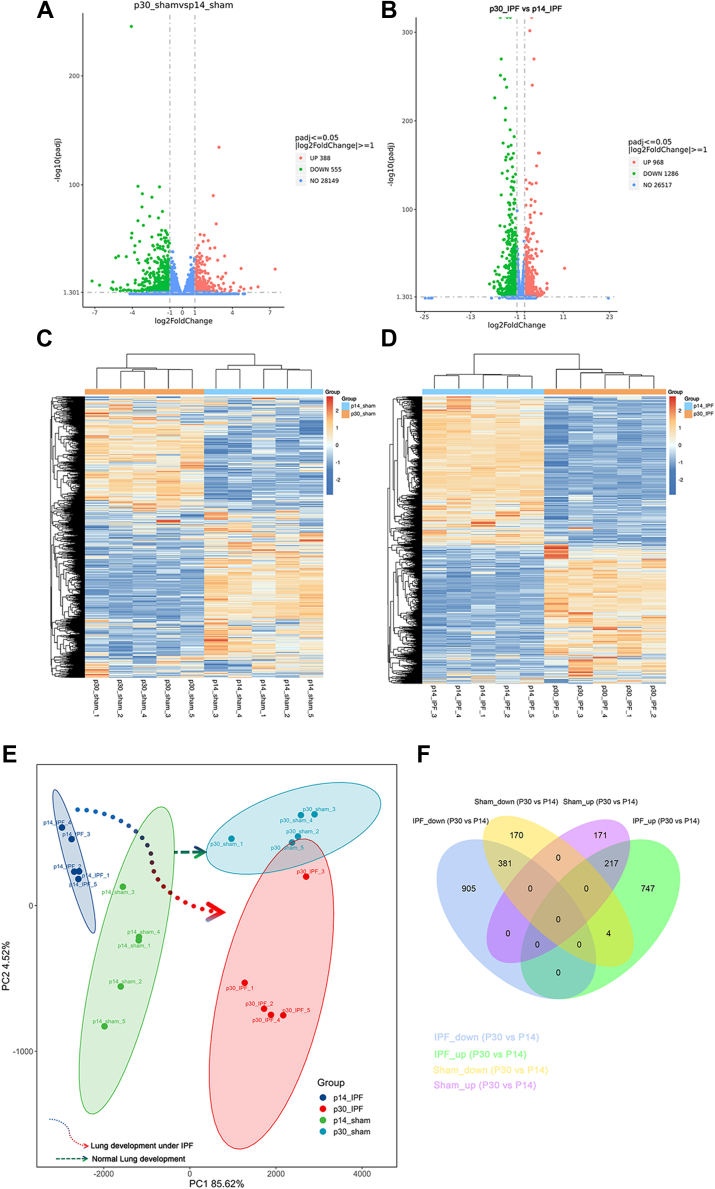
Postnatal Lung Maturation Is Changed by IPF (A) DEG volcano plot: P30_Sham vs P14_Sham (normal maturation). (B) DEG volcano plot: P30_IPF vs P14_IPF (IPF-affected maturation). (C and D) Heatmaps of DEGs in (C) normal and (D) IPF-affected maturation groups. (E) Principal component analysis showing transcriptomic divergence (greater P14_IPF→P30_IPF distance vs P14_Sham→P30_Sham). (F) Venn diagram: Shared/unique DEGs between normal and IPF-affected maturation. DEG = differentially expressed gene.

To investigate how IPF reshapes postnatal lung maturation, lung tissues at P14 (ALV4 stage) and P30 (mature lung stage) were selected for RNA-seq analysis. The volcano plot revealed 943 DEGs in normal lung maturation (P30_Sham vs P14_Sham), with 388 upregulated and 555 downregulated (Figure 2A). In contrast, there were 2,272 DEGs in IPF-disrupted lung maturation (P30_IPF vs P14_IPF), with 968 upregulated and 1,286 downregulated (Figure 2B), indicating that IPF increases the number of DEGs. The heatmap of DEGs showed high similarity within the same group but significant differences between groups in both normal and IPF-disrupted lung maturation (Figures 2C to 2D).

**Figure 3:**
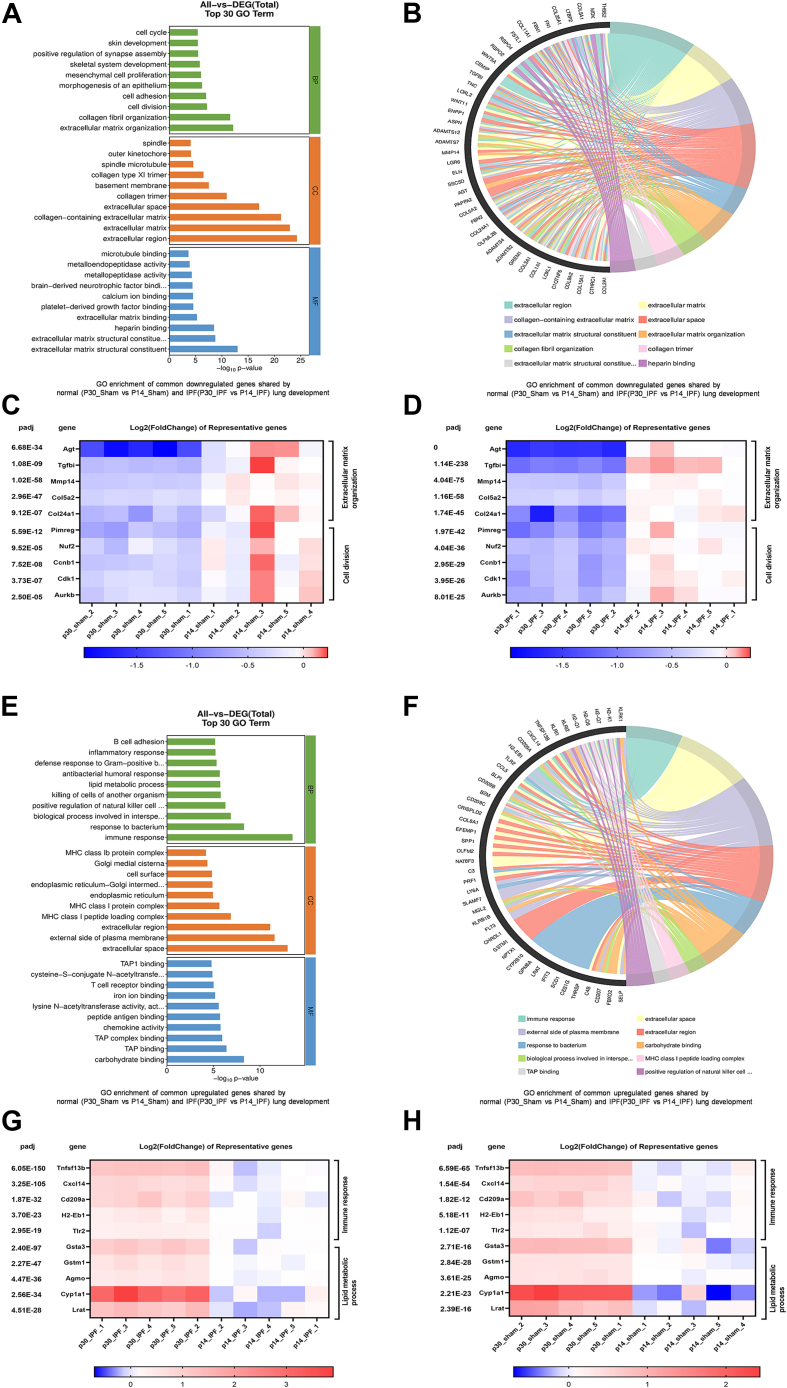
Unchanged Biological Events of Normal Lung Maturation Under IPF Influence (A) The top 30 terms of gene ontology (GO) enrichment analysis of the shared downregulated DEGs between normal and IPF-disrupted lung maturation. (B) The chord diagram of GO enrichment analysis of the shared downregulated DEGs. (C) Heatmap of representative genes associated with cell cycle and extracellular matrix organization in normal lung maturation. (D) Heatmap of representative genes associated with cell cycle and extracellular matrix organization in IPF-disrupted lung maturation. (E) The top 30 terms of GO enrichment analysis of the shared upregulated DEGs between normal and IPF-disrupted lung maturation. (F) The chord diagram of GO enrichment analysis of the shared upregulated DEGs. (G) Heatmap of representative genes associated with immune responses in normal lung maturation. (H) Heatmap of representative genes associated with immune responses in IPF-disrupted lung maturation. BP, biological process; CC, cellular component; MF, molecular function; other abbreviations as in Figures 1 and 2.

Principal component analysis of DEGs demonstrated distinct transcriptomic profiles among the 4 groups, with the distance from P14_IPF to P30_IPF significantly longer than that from P14_Sham to P30_Sham (Figure 2E), suggesting that IPF alters the trajectory of postnatal lung maturation.

To further explore how IPF affects postnatal lung maturation, a Venn diagram analysis was performed to identify shared DEGs between normal and IPF-disrupted lung maturation, as well as unique DEGs for each condition. The results showed that normal and IPF-disrupted lung maturation shared 598 DEGs, with 381 downregulated and 217 upregulated. There were 341 unique DEGs in normal lung maturation, with 170 downregulated and 171 upregulated, and 1,652 unique DEGs in IPF-disrupted lung maturation, with 905 downregulated and 747 upregulated (Figure 2F).

### Unchanged biological events of normal lung maturation under IPF influence

To identify biological events in normal lung maturation that remain unchanged under IPF, shared DEGs were subjected to GO enrichment analysis.

As shown in Figure 3A, the top 10 enriched GO terms for the shared 381 downregulated DEGs in biological process, cellular component, and molecular function were primarily associated with extracellular matrix (ECM) organization and cell cycle. The chord diagram further highlighted ECM organization as a key pathway (Figure 3B). The heatmap of representative ECM- and cell cycle–associated genes in the top 10 GO terms confirmed their downregulation in both normal and IPF-disrupted lung maturation (Figures 3C and 3D). Given that alveolar formation and single-layer capillary network development are closely linked to ECM remodeling and cell cycle regulation,^1^^,^^2^ these results suggest that lung maturation progresses under IPF, albeit at a reduced rate (Figure 2E).

As shown in Figure 3E, the top 10 enriched GO terms for the shared 217 upregulated DEGs in biological process, cellular component, and molecular function were primarily associated with immune responses. The chord diagram also highlighted immune response pathways (Figure 3F). The heatmap of representative immune response–associated genes confirmed their upregulation in both normal and IPF-disrupted lung maturation (Figures 3G and 3H). These findings reveal a previously unrecognized aspect of lung maturation—immune response maturation—which remains unaffected by IPF.

### Disruption of key biological processes in normal lung maturation by IPF

To delineate biological events integral to normal lung maturation that are disrupted by IPF, unique DEGs of normal lung maturation were subjected to GO and KEGG pathway enrichment analysis.

**Figure 4:**
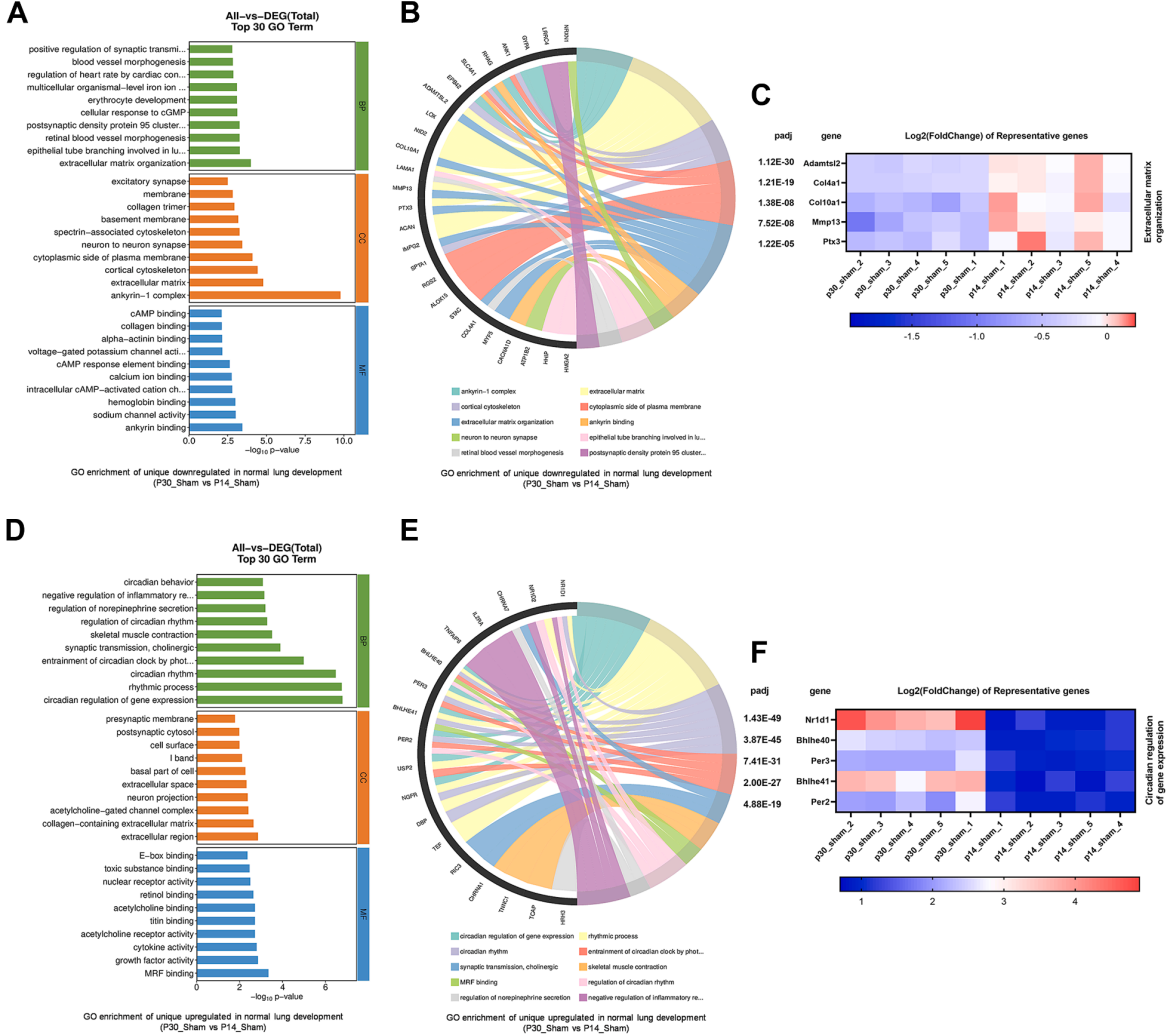
Disrupted Biological Events of Normal Lung Maturation Under IPF Influence (A) The top 30 terms of GO enrichment analysis of the unique downregulated DEGs in normal lung maturation. (B) The chord diagram of GO enrichment analysis of the unique downregulated DEGs. (C) Heatmap of representative genes associated with ECM organization in normal lung maturation. (D) The top 30 terms of GO enrichment analysis of the unique upregulated DEGs in normal lung maturation. (E) The chord diagram of GO enrichment analysis of the unique upregulated DEGs. (F) Heatmap of representative genes associated with circadian regulation in normal lung maturation. ECM = extracellular matrix; other abbreviations as in Figure 1, Figure 2, Figure 3.

As shown in Figure 4A, the top 10 GO terms for the unique 170 downregulated DEGs in normal lung maturation were associated with ECM organization, epithelial tube branching in lung morphogenesis, blood vessel morphogenesis, and regulation of synaptic transmission. The chord diagram highlighted blood vessel morphogenesis and neuron-to-neuron synapse pathways (Figure 4B). The heatmap of representative ECM-associated genes confirmed their downregulation in normal lung maturation (Figure 4C). Complementary KEGG pathway analysis corroborated these findings, showing enrichment in pathways crucial for alveolar maturation (eg, Wnt signaling, Axon guidance), capillary maturation (eg, Apelin signaling pathway, apoptosis), and neural regulation (eg, neuroactive ligand-receptor interaction, synaptic vesicle cycle) (Supplemental Figure 1A). Collectively, these results suggest more profound remodeling associated with alveolar maturation, single-layer capillary formation, and neural regulation maturation in normal lung maturation.

As shown in Figure 4D, the top 10 GO terms for the unique 171 upregulated DEGs in normal lung maturation were associated with circadian rhythm. The chord diagram also highlighted circadian rhythm pathways (Figure 4E). The heatmap of representative circadian rhythm–associated genes confirmed their upregulation in normal lung maturation (Figure 4F). Supporting KEGG pathway analysis demonstrated enrichment specifically in circadian rhythm pathways (eg, circadian rhythm, circadian entrainment) (Supplemental Figure 1B). These findings uncover a previously unrecognized aspect of postnatal lung maturation—circadian rhythm maturation—which is disrupted by IPF. Furthermore, the data strongly suggest that neural regulation maturation may be severely impaired by IPF.

### Novel and emerging biological events in IPF-disrupted lung maturation

To delineate novel biological events in IPF-disrupted lung maturation, unique DEGs of IPF-disrupted lung maturation were subjected to GO enrichment analysis.

**Figure 5:**
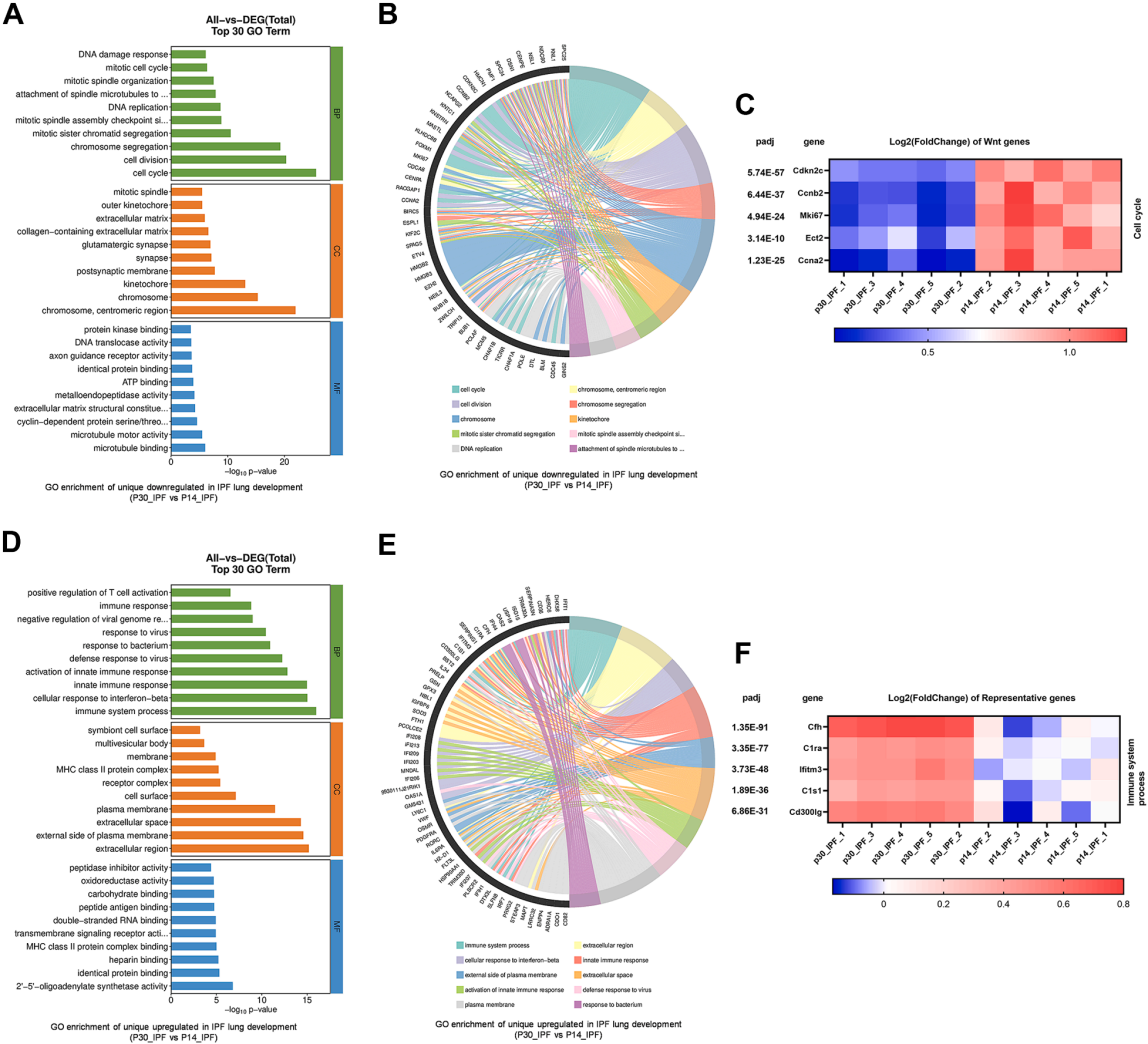
Novel and Emerging Biological Events in IPF-Disrupted Lung Maturation (A) The top 30 terms of GO enrichment analysis of the unique downregulated DEGs in IPF-disrupted lung maturation. (B) The chord diagram of GO enrichment analysis of the unique downregulated DEGs. (C) Heatmap of representative genes associated with cell cycle in IPF-disrupted lung maturation. (D) The top 30 terms of GO enrichment analysis of the unique upregulated DEGs in IPF-disrupted lung maturation. (E) The chord diagram of GO enrichment analysis of the unique upregulated DEGs. (F) Heatmap of representative genes associated with immune responses. Abbreviations as in Figure 1, Figure 2, Figure 3.

As shown in Figure 5A, the top 10 GO terms for the unique 905 downregulated DEGs in IPF-disrupted lung maturation were associated with cell cycle. The chord diagram also highlighted cell cycle pathways (Figure 5B). The heatmap of representative cell cycle–associated genes confirmed their downregulation in IPF-disrupted lung maturation (Figure 5C). Complementary KEGG pathway analysis corroborated this finding, demonstrating enrichment in core cell cycle pathways (eg, homologous recombination, cell cycle) (Supplemental Figure 1C). Collectively, these results suggest that IPF induces heightened cell cycle activity from the ALV4 to mature lung stage, possibly due to alveolar dysplasia at ALV4, which triggers cell proliferation not only in alveolar epithelial cells but also in pulmonary vascular smooth muscle cells (VSMCs), fibroblasts, and immune cells.

As shown in Figure 5D, the top 10 GO terms for the unique 747 upregulated DEGs in IPF-disrupted lung maturation were associated with immune responses. The chord diagram also highlighted immune response pathways (Figure 5E). The heatmap of representative immune response–associated genes confirmed their upregulation in IPF-disrupted lung maturation (Figure 5F). Supporting KEGG pathway analysis confirmed enrichment in key immune and inflammatory pathways (eg, complement and coagulation cascades, antigen processing and presentation) (Supplemental Figure 1D). These findings demonstrate that inflammatory immunity is abnormally activated in IPF-disrupted lung maturation.

### Validation of RNA-seq results

To validate the RNA-seq results, we examined alveolar maturation, microcirculation maturation, and remodeling of pulmonary small blood vessels.

**Figure 6:**
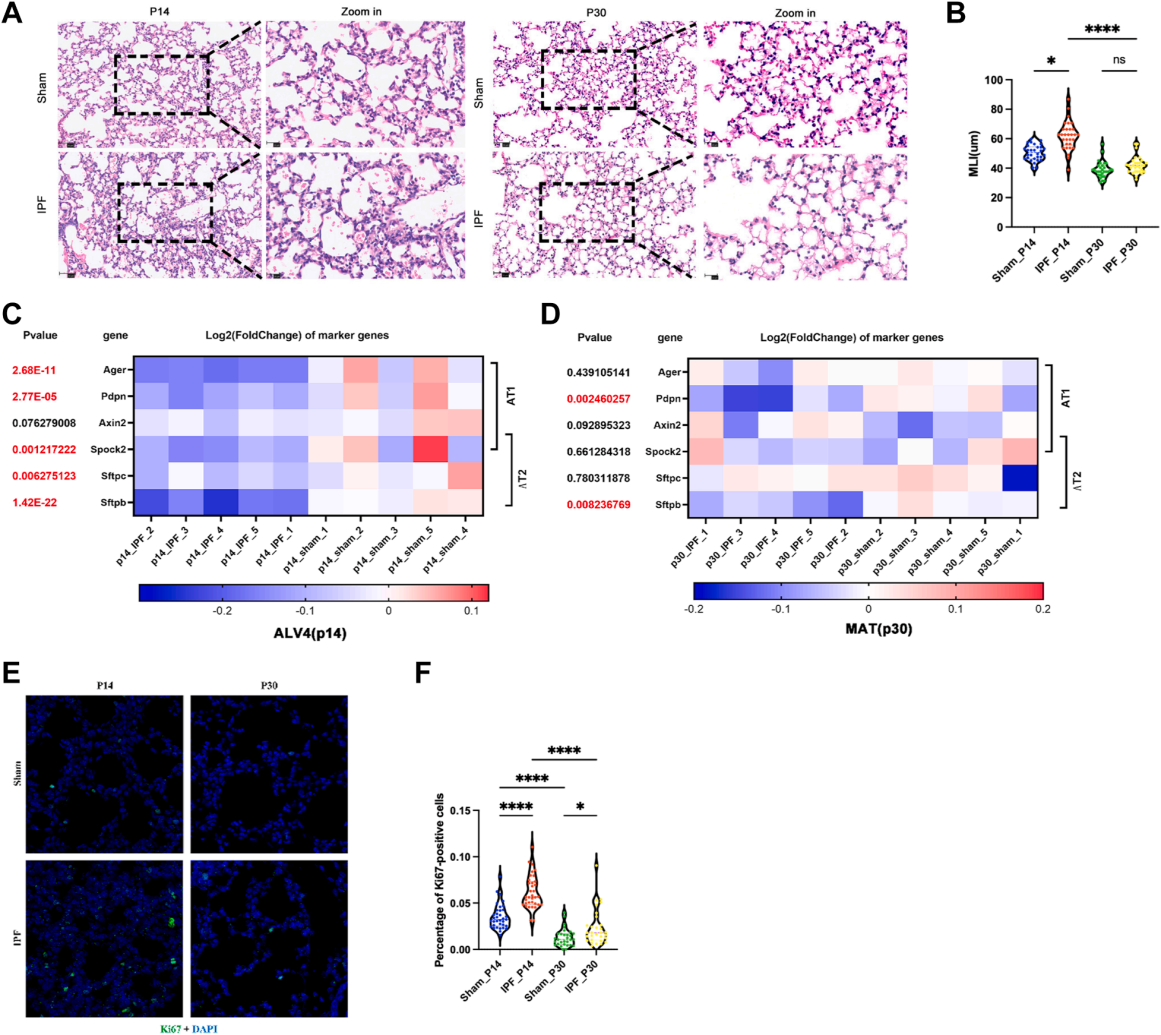
Verification of RNA-seq Analysis Via Alveolar Maturation Examination (A) Representative H&E staining of alveolus at P14 and P30. (B) Quantification of mean linear intercept (MLI). The larger the MLI value, the fewer alveoli there are. (C) Heatmap of AT1 and AT2 marker genes at P14. (D) Heatmap of AT1 and AT2 marker genes at P30. (E) Representative Ki67 staining at P14 and P30. Ki67 (green); DAPI (blue). (F) Quantification of Ki67-positive cells. ∗*P* < 0.05, ∗∗∗∗*P* < 0.0001, and ns, not significant. AT1/AT2 = alveolar type 1/type 2 epithelial cells; DAPI = 4′,6-diamidino-2-phenylindole; other abbreviations as in Figure 1.

As shown in Figures 6A and 6B, at P14 (ALV4 stage), the number of alveoli in the IPF group was significantly lower than in the sham group. However, from P14 to P30, the number of alveoli in the IPF group continued to increase, although it remained lower than in the sham group at P30. These results align with the GO enrichment analysis of shared downregulated DEGs, which are primarily associated with ECM organization and cell cycle (Figures 3A to 3D). Notably, the reduction in alveolar number in the IPF group at P30 was less pronounced than at P14, indicating a higher alveolar growth rate in the IPF group from P14 to P30, consistent with the GO enrichment analysis of IPF-unique downregulated DEGs associated with cell cycle (Figures 5A to 5C). The heatmap of alveolar type 1/type 2 epithelial cells (AT1/AT2) marker genes, along with their quantitative reverse transcriptase polymerase chain reaction results, showed greater differences in expression levels between the IPF and sham groups at P14 than at P30 (Figure 6C and 6D, Supplemental Figures 2A and 2B), further supporting the alveolar quantity analysis (Figures 6A and 6B). In addition, we observed a significant increase in the proliferation marker Ki67 in IPF lungs compared with sham lungs at P14 (Figures 6E and 6F), with the percentage of Ki67-positive cells gradually decreasing from P14 to P30 in both groups (Figures 6E and 6F).

**Figure 7:**
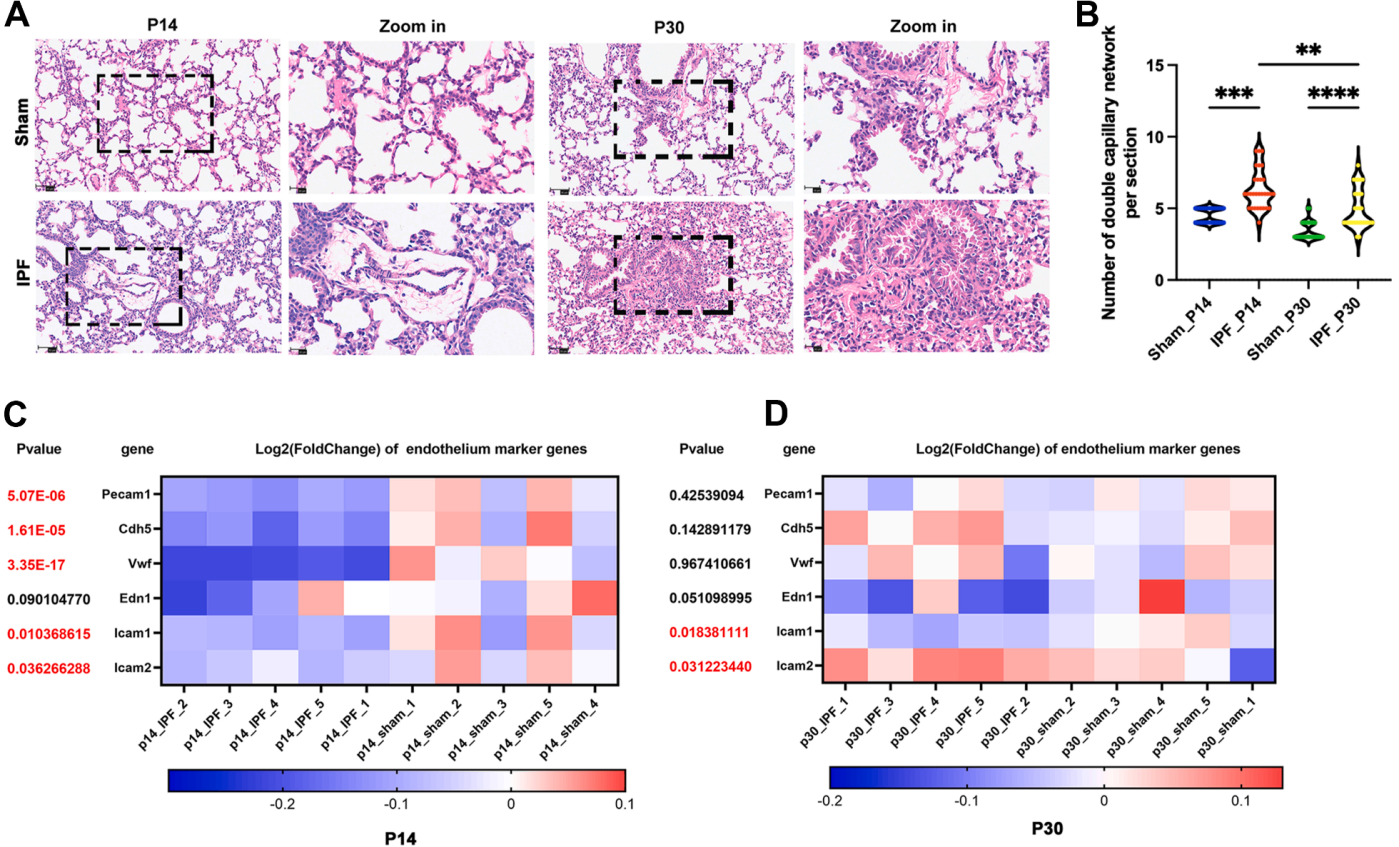
Verification of RNA-seq Analysis Via Microcirculation Maturation Examination (A) Representative H&E staining of double capillaries at P14 and P30. (B) Quantification of the number of double capillaries at P14 and P30. (C) Heatmap of endothelium-marker genes at P14. (D) Heatmap of endothelium-marker genes at P30. ∗∗*P* < 0.01, ∗∗∗*P* < 0.001, ∗∗∗∗*P* < 0.0001. Abbreviations as in Figure 1.

Next, we examined microcirculation maturation. As shown in Figures 7A and 7B, at P14 (ALV4 stage), the IPF group exhibited more double capillary networks in the parenchymal septa than the sham group. These networks decreased from P14 to P30 in both groups, but the IPF group still had more double capillary networks at P30. The reduction in single capillary networks in the IPF group, analogous to the reduction in alveolar quantity, was consistent with the RNA-seq results. The heatmap of vascular endothelial marker genes along with their quantitative reverse transcriptase polymerase chain reaction results showed greater differences in expression levels between the IPF and sham groups at P14 than at P30 (Figures 7C and 7D, Supplemental Figures 2C and 2D), further validating the single capillary network analysis (Figures 7A and 7B).

**Figure 8:**
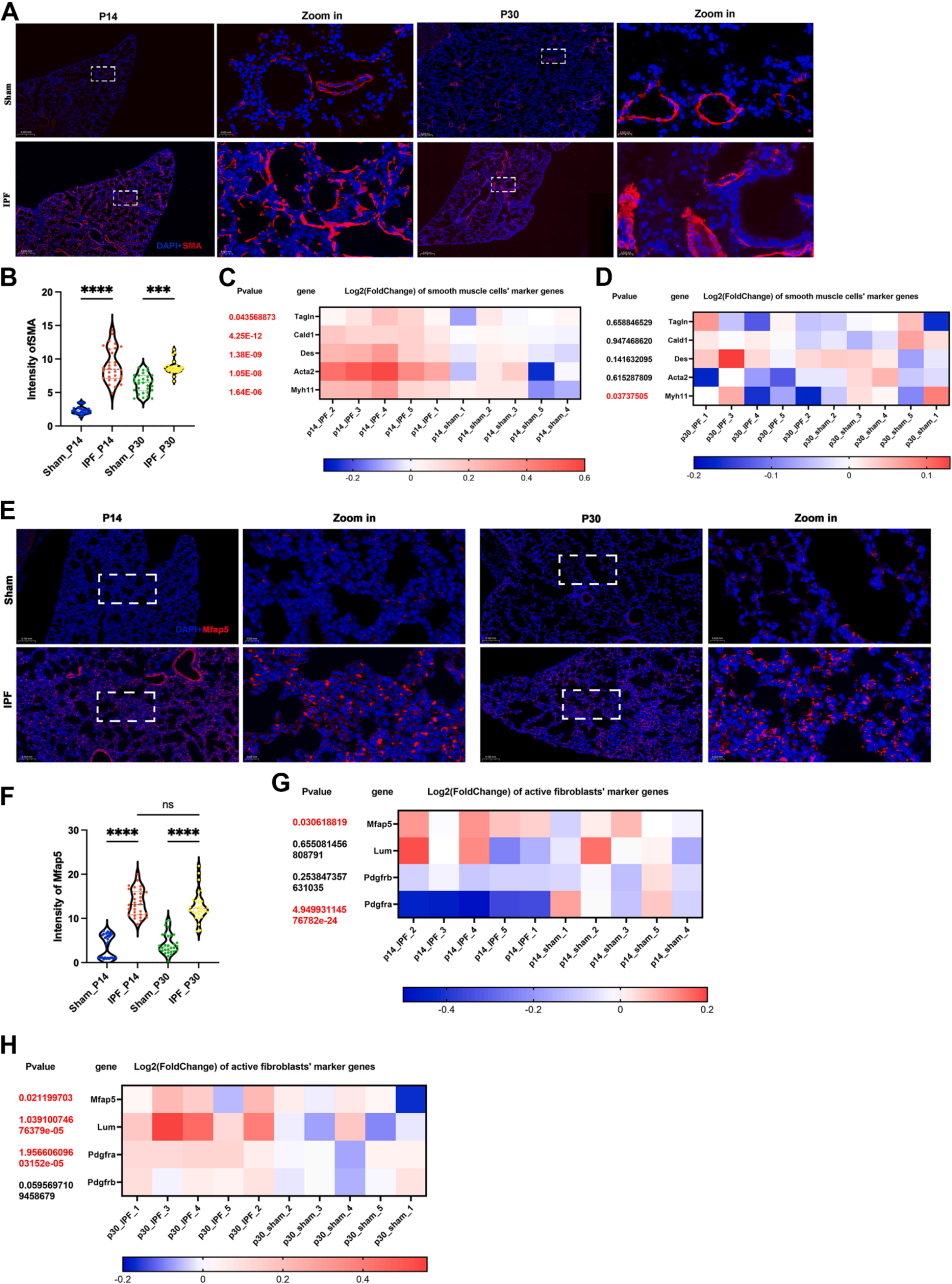
Verification of RNA-seq Via PH-Related Marker Analysis (A) Representative smooth muscle actin (SMA) staining at P14 and P30; SMA (red); DAPI (blue). (B) Quantification of fluorescence intensity of SMA. (C) Heatmap of smooth muscle cells’ marker genes at P14. (D) Heatmap of smooth muscle cells’ marker genes at P30. (E) Representative active fibroblasts (Mfap5) staining at P14 and P30; Mfap5 (red); DAPI (blue). (F) Quantification of fluorescence intensity of Mfap5. (G) Heatmap of active fibroblasts’ marker genes at P14. (H) Heatmap of active fibroblasts’ marker genes at P30. ∗∗∗*P* < 0.001, ∗∗∗∗*P* < 0.0001 and ns, not significant. Abbreviations as in Figures 1 and 6.

Finally, we examined markers of PH, including increased expression of VSMCs and active fibroblasts (characterized by active ECM production).^37^^,^^38^ As shown in Figures 8A and 8B, immunostaining revealed significantly higher VSMC expression in the IPF group than in the sham group at both P14 and P30, with no further increase from P14 to P30. At the messenger RNA (mRNA) level, all VSMC marker genes were significantly upregulated in the IPF group at P14 (Figure 8C, Supplemental Figure 2E). By P30, these marker genes showed no significant differences between the 2 groups, with myh11 even downregulated (Figure 8D, Supplemental Figure 2F). Similarly, immunostaining showed significantly higher active fibroblast expression in the IPF group than in the sham group at both P14 and P30, with no further increase from P14 to P30 (Figures 8E and 8F). At the mRNA level, only Mfap5 was significantly upregulated in the IPF group at P14 (Figure 8G, Supplemental Figure 2G). By P30, marker genes for active fibroblasts were increased (Figure 8H, Supplemental Figure 2H). These results indicate that VSMCs and active fibroblasts undergo active changes from P14 to P30, consistent with the RNA-seq enrichment analysis (Figures 3A to 3D).

### Immune activation drives lung maturation disruption in IPF

Although the enrichment of immune-related pathways in IPF-affected lungs is a key finding, it raises a critical question regarding causality: is immune activation a primary driver of disrupted lung maturation or merely a secondary consequence of vascular/mechanical stress?

**Figure 9:**
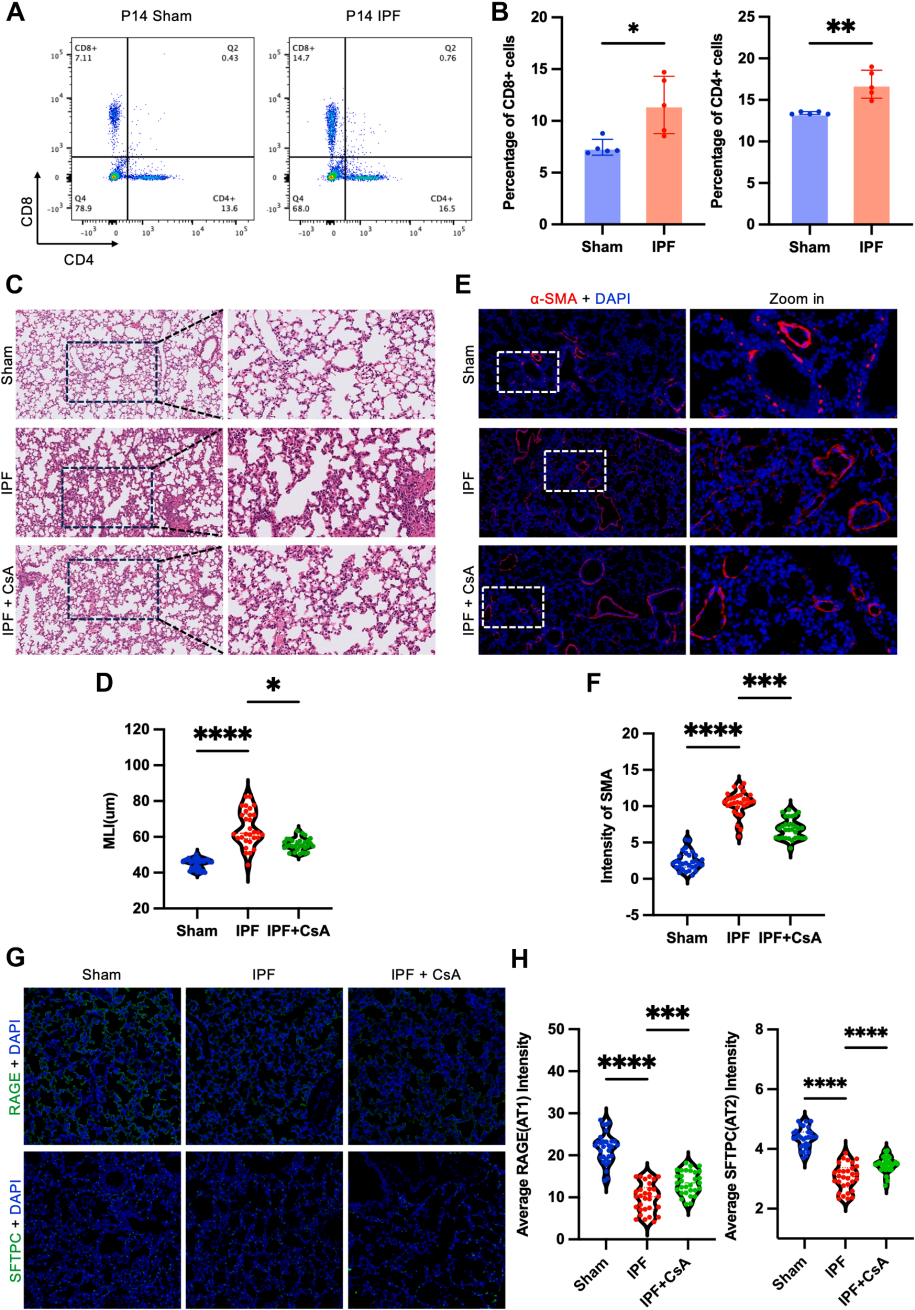
CsA Attenuates IPF Vasculopathy (A) Representative flow cytometry plots of CD4+/CD8+ cells from sham and IPF groups. (B) Quantification of the percentage of CD4+, CD8+ cells. (C) Representative H&E staining of lung tissues from sham, IPF, or CsA-treated groups. (D) Quantification of MLI. (E) Representative of SMA staining from IPF and CsA-treated groups; SMA (red); DAPI (blue). (F) Quantification of fluorescence intensity of SMA. (G) Representative alveolar type 1 (AT1) and alveolar type 2 (AT2) cells from sham, IPF, and CsA-treated lungs. Rage (green); Sftpc (green); DAPI (blue). (H) Quantification of Rage and Sftpc intensity. ∗*P* < 0.05, ∗∗*P* < 0.01, ∗∗∗*P* < 0.001, ∗∗∗∗*P* < 0.0001. CsA = cyclosporine A; other abbreviations as in Figure 1, Figure 6, and 8.

To address this, we first quantified CD4^+^ and CD8^+^ T cells in lung tissue at P14. This analysis revealed a significant increase in both CD4^+^ and CD8^+^ T-cell populations in IPF lungs compared with sham controls (Figures 9A and 9B), confirming early immune involvement coinciding with the active alveolarization phase. Next, to functionally assess the role of T-cell activation, we administered CsA—a potent T-cell inhibitor—using a previously established regimen.^31^ CsA treatment was initiated during the critical alveolarization window, before the peak of pathological manifestations. Strikingly, CsA administration significantly ameliorated key pathological features: it reduced alveolar simplification (Figures 9C and 9D), attenuated vascular remodeling (Figures 9E and 9F), and improved alveolar epithelial cell differentiation (Figures 9G and 9H).

The preventive efficacy of CsA, achieved when administered prophylactically before significant pathology develops, provides compelling evidence that immune activation is not simply a passive secondary response to mechanical stress.

## Discussion

IPF, one of the most critical hemodynamic abnormalities in pediatric cardiovascular pathologies, often arises from congenital heart diseases with left-to-right shunts. It has long been recognized to exert serious adverse effects on pediatric cardiopulmonary functions over time, including PH and respiratory distress.^1^, ^2^, ^3^, ^4^, ^5^ However, because of the absence of a neonatal IPF animal model, there have been no reports on how IPF reshapes postnatal lung maturation to date. In 2021, the first neonatal mouse model of ACF surgery, which induces a left-to-right shunt, was reported, revealing how this shunt alters postnatal right ventricular development.^27^ In contrast, this study introduces the first neonatal mouse model of IPF via ACF surgery. This model provides a robust platform for future investigations into the molecular and cellular mechanisms underlying IPF and its associated complications. It can be used to test potential therapies and further explore the role of specific cell types, such as mesenchymal (Mfap5+) cells,^22^^,^^39^ in lung development.

Using bulk RNA-seq, the study reveals distinct transcriptomic profiles between normal and IPF-disrupted lung maturation (Central Illustration). It identifies shared and unique DEGs and their associated biological processes, such as ECM organization, cell cycle regulation, immune responses, and circadian rhythm. The study uncovers key changes in alveolar development, microcirculation, neural regulation, and immune responses, providing novel insights into the mechanisms underlying IPF-associated lung pathology. In summary, this study is the first to comprehensively investigate how IPF alters postnatal lung maturation at the molecular level, significantly expanding our understanding of postnatal lung development and highlighting the complexity of this process. By identifying key pathways and genes involved in IPF-disrupted lung maturation, the study opens new avenues for therapeutic interventions.

**Central Illustration:**
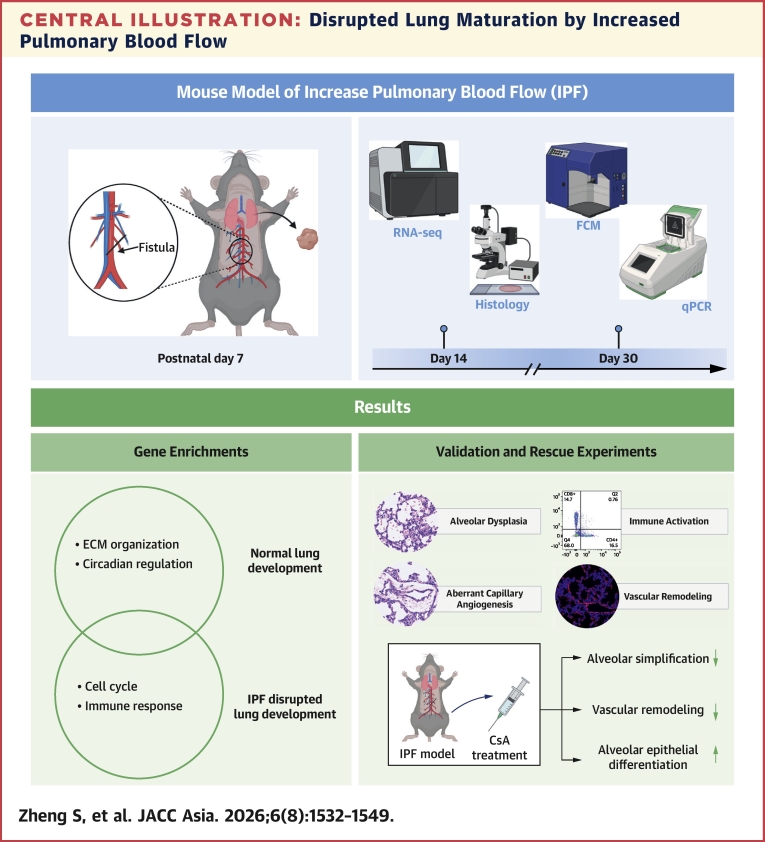
Disrupted Lung Maturation by Increased Pulmonary Blood Flow This central illustration summarizes how IPF disrupts postnatal lung maturation and its clinical implications. CsA = cyclosporine A; ECM = extracellular matrix; FCM = flow cytometry; IPF = increase pulmonary blood flow; qPCR = quantitative polymerase chain reaction; RNA-seq = RNA sequencing.

For instance, targeting mesenchymal (Mfap5+) cells or neural regulation^40^ could potentially mitigate the adverse effects of IPF on lung development, such as pediatric PH and alveolar dysplasia. Indeed, mesenchymal cells, which transiently appear during alveolar maturation and are absent in adult animals, have been shown to play a crucial role in alveolar maturation.^19^^,^^20^ Furthermore, Mfap5+ cells have been demonstrated to be critical for ECM production.^39^^,^^41^ Given the elevated expression of Mfap5 in mesenchymal cells, future studies should investigate the role of these cells in alveolar development and repair, potentially through single-cell RNA-seq or lineage-tracing experiments.

Our study further elucidates the pathological role of immune activation in PH—a mechanism increasingly implicated in PH pathogenesis. Although T-cell–mediated immunity has been established as a critical driver of vascular remodeling in PH,^42^ we extend this paradigm by demonstrating: 1) pronounced enrichment of immune pathways in IPF-induced pediatric PH and; 2) functional validation through CsA intervention, which significantly attenuated pathological vascular remodeling and alveolar simplification. These insights critically advance the field by providing direct experimental evidence for immune dysregulation in hemodynamically driven pediatric pulmonary arterial hypertension and establishing the therapeutic potential of early immunomodulation. This mechanistic foundation strongly supports targeted immune therapies for early-stage disease.

A critical translational consideration is the extent to which our murine model recapitulates human pediatric IPF-PH pathophysiology. As delineated in Supplemental Table 2, the model faithfully mirrors core pathological features observed in early human disease—particularly ECM disorganization and immune dysregulation—while also uncovering developmentally arrested mechanisms unique to postnatal lung maturation (eg, alveolar maturation arrest, neural/circadian axis disruption). This intersection of conserved and ontogeny-specific pathways underscores that effective pediatric interventions must target transient developmental cell populations (eg, MFAP5^+^ fibroblasts) and stage-specific signaling hubs (eg, circadian entrainment).

### Study limitations

#### Mechanistic depth

Although our transcriptomic analyses identify IPF-associated dysregulation of immune activation (NLRP3/IL23R) and cell cycle control (Birc5/CENPE), the intrinsic signaling cascades linking hemodynamic stress to these molecular changes remain incompletely delineated. Future studies should employ genetic/pharmacological perturbations to establish causal mechanisms.

#### Repair mechanisms

Although we observed substantial resolution of alveolar simplification by P30, the molecular drivers enabling this compensatory repair—particularly in ECM reorganization and progenitor cell activation—are undefined. Elucidating these pathways could reveal regenerative targets for childhood IPF.

#### Translational relevance

Despite phenotypic concordance in early pathology (Supplemental Table 2), ontogenetic differences in lung development timelines between mice and humans necessitate caution. Validating key targets (eg, MFAP5^+^ fibroblasts, circadian regulators) in pediatric patient samples is essential before clinical translation.

## Conclusions

In summary, this study represents a significant advancement in our understanding of how IPF affects postnatal lung maturation. By introducing a novel neonatal mouse model and providing comprehensive transcriptomic insights, the study sheds light on the molecular mechanisms underlying IPF-associated lung pathology. Despite its limitations, the findings open new avenues for therapeutic interventions and future research, with the potential to improve outcomes for children with IPF-associated diseases.

## Funding Support and Author Disclosures

This work was supported by the National Natural Science Foundation of China (82270314 and 82400363), the Ningbo Top Medical and Health Research Program (no. 2022020405), the Science and Technology Development Fund of Pudong New Area (PKJ2024-Y13, PKJ2024-Y14, and PKJ2024-Y15), the National Clinical Key Specialty Construction Project (10000015Z155080000004), Shanghai Key Research Center Construction Project-Shanghai Research Center for Pediatric Cardiovascular Diseases (2023ZZ02024), the Medical and Health Technology Plan Projects of Zhejiang (2024YK1568), and the Ningbo Medical and Health Brand Discipline (PPXK2024-06). The authors have reported that they have no relationships relevant to the contents of this paper to disclose.
