# Supplementary material for: The Postnatal Lung Maturation Disrupted by Increased Pulmonary Blood Flow and Its Clinical Implications
Source: JACC Asia. 2026 Mar 26;6(8):1532–49. doi: 10.1016/j.jacasi.2026.02.013 (PMC13458924; doi:10.1016/j.jacasi.2026.02.013)
Supplement: Supplementary file 1 [file mmc1.docx]

**Supplemental Figure 1 KEGG pathway enrichment analysis of unique DEGs in normal and IPF-Disrupted lung maturation.**

(A) Top 20 enrichment of unique downregulated DEGs in normal lung development. (B) Top 20 enrichment of unique upregulated DEGs in normal lung development. (C) Top 20 enrichment of unique downregulated DEGs in IPF lung development. (D) Top 20 enrichment of unique upregulated DEGs in IPF lung development.

**Supplemental Figure 2 Verification of RNA-seq results by qRT-PCR**

(A)The mRNA level of alveolar epithelial marker genes at P14. (B) The mRNA level of alveolar epithelial marker genes at P30. (C)The mRNA level of endothelial marker genes at P14. (D)The mRNA level of endothelial marker genes at P30. (E)The mRNA level of VSMC marker genes at P14. (F)The mRNA level of VSMC marker genes at P30. (G)The mRNA level of fibroblast marker genes at P14. (H)The mRNA level of fibroblast marker genes at P30. *p<0.05, **p<0.01, ***p<0.001, ****p<0.0001, ns=no significance

**Supplemental Table 1 Primer sequences used for qPCR validation**

| Gene |  | Sequence (5'->3') |
| --- | --- | --- |
| Arge | Forward | CTTGCTCTATGGGGAGCTGTA |
|  | Reverse | GGAGGATTTGAGCCACGCT |
| Pdpn | Forward | ACCGTGCCAGTGTTGTTCTG |
|  | Reverse | AGCACCTGTGGTTGTTATTTTGT |
| Axin2 | Forward | TGACTCTCCTTCCAGATCCCA |
|  | Reverse | TGCCCACACTAGGCTGACA |
| Spock2 | Forward | ACCCCCGGCAATTTCATGG |
|  | Reverse | TGTCTTCCCAGCTCTTGATGTAA |
| Sftpc | Forward | ATGGACATGAGTAGCAAAGAGGT |
|  | Reverse | CACGATGAGAAGGCGTTTGAG |
| Sftpb | Forward | CTGCTTCCTACCCTCTGCTG |
|  | Reverse | CTTGGCACAGGTCATTAGCTC |
| Pecam1 | Forward | ACGCTGGTGCTCTATGCAAG |
|  | Reverse | TCAGTTGCTGCCCATTCATCA |
| Cdh5 | Forward | CACTGCTTTGGGAGCCTTC |
|  | Reverse | GGGGCAGCGATTCATTTTTCT |
| Vwf | Forward | CTTCTGTACGCCTCAGCTATG |
|  | Reverse | GCCGTTGTAATTCCCACACAAG |
| Edn1 | Forward | GCACCGGAGCTGAGAATGG |
|  | Reverse | GTGGCAGAAGTAGACACACTC |
| Icam1 | Forward | GTGATGCTCAGGTATCCATCCA |
|  | Reverse | CACAGTTCTCAAAGCACAGCG |
| Icam2 | Forward | CACAGTTCTCAAAGCACAGCG |
|  | Reverse | GAGGCTGGTACACCCTGATG |
| Tagln | Forward | CAACAAGGGTCCATCCTACGG |
|  | Reverse | ATCTGGGCGGCCTACATCA |
| Cald1 | Forward | ATGGTAGAGGAGAAAACACCAGA |
|  | Reverse | CCATCCCCTTCTATTTTGGACTC |
| Des | Forward | GTGGATGCAGCCACTCTAGC |
|  | Reverse | TTAGCCGCGATGGTCTCATAC |
| Acta2 | Forward | GTCCCAGACATCAGGGAGTAA |
|  | Reverse | TCGGATACTTCAGCGTCAGGA |
| Myh11 | Forward | AAGCTGCGGCTAGAGGTCA |
|  | Reverse | CCCTCCCTTTGATGGCTGAG |
| Mfap5 | Forward | GTCTTGGCAATCAGCATCCC |
|  | Reverse | CCAGATTAGGGTCGTCTGTGAAT |
| Lum | Forward | CTCTTGCCTTGGCATTAGTCG |
|  | Reverse | GGGGGCAGTTACATTCTGGTG |
| Pdgfrb | Forward | TTCCAGGAGTGATACCAGCTT |
|  | Reverse | AGGGGGCGTGATGACTAGG |
| Pdgfra | Forward | TCCATGCTAGACTCAGAAGTCA |
|  | Reverse | TCCCGGTGGACACAATTTTTC |

**Supplemental Table 2 Pathological Characteristics of human pediatric IPF-PAH vs. Mouse IPF Model (Focus: ECM & Immunity)**

|  | human pediatric IPF-PAH* | Mouse IPF Model | Overlap/Divergence |
| --- | --- | --- | --- |
| 1. Extracellular Matrix (ECM) Remodeling |  |  |  |
| ECM Composition | ↑ Collagen I/III, elastin, fibronectin in pulmonary arteries | ↓ ECM organization genes (e.g., Col1a1, Eln) | Both show ECM dysregulation, but human IPF -PAH emphasizes vascular fibrosis, while Mouse IPF Model shows global alveolar-capillary ECM impairment. |
| Cellular Drivers | - Hyperplastic SMCs  -Activated fibroblasts (MFAP5+)  -Endothelial-mesenchymal transition | -Proliferating fibroblasts (↑ Mfap5)  -Dysregulated AT1/AT2 cells  -↑ Ki67+ cells | Shared: Fibroblast activation (MFAP5+) drives fibrosis.  Divergence: IPF shows broader alveolar cell cycle dysregulation. |
| Structural Outcome | - Medial hypertrophy  - Plexiform lesions  - Arterial stiffening | - Alveolar simplification (↓ alveoli count)  - Delayed capillary maturation (persistent double-capillary networks) | Both exhibit impaired vascular remodeling, but IPF uniquely disrupts developmental alveolar septation. |
| 2. Immune Dysregulation |  |  |  |
| Immune Activation | Macrophage/T-cell infiltration  - ↑ IL-6, TNF-α, TGF-β  - Autoantibodies (e.g., anti-EC) | - ↑ Neutrophil/innate immune genes  - ↑ MHC class II, chemokines (e.g., Cxcl1)  - Persistent inflammation | Shared: Chronic inflammation amplifies remodeling.  Divergence: Human PAH involves adaptive immunity; IPF shows acute neonatal innate responses. |
| Impact on Remodeling | Immune cells promote:  - SMC proliferation  - Fibroblast activation  - ECM deposition | Immune genes correlate with:  - Alveolar dysplasia  - Delayed microcirculation maturation | Both link immunity to fibrosis progression, but IPF ties inflammation to arrested development. |

*references

1. van der Feen DE, Bartelds B, de Boer RA, Berger RMF. Pulmonary arterial hypertension in congenital heart disease: translational opportunities to study the reversibility of pulmonary vascular disease. Eur Heart J. 2017 Jul 7;38(26):2034-2041. doi: 10.1093/eurheartj/ehx034. PMID: 28369399.
2. Klouda T, Tsikis ST, Hirsch TI, Kim Y, Liu T, Friehs I, Shyy JY, Visner G, Raby BA, Puder M, Yuan K. Smooth muscle Cxcl12 activation is associated with vascular remodeling in flow-induced pulmonary hypertension. bioRxiv [Preprint]. 2024 Sep 10:2024.09.10.611870. doi: 10.1101/2024.09.10.611870. Update in: J Biol Chem. 2025 May 8;301(6):110207. doi: 10.1016/j.jbc.2025.110207. PMID: 39314465; PMCID: PMC11418988.
3. Jančauskaitė D, Rudienė V, Jakutis G, Geenen LW, Roos-Hesselink JW, Gumbienė L. Residual Pulmonary Hypertension more than 20 Years after Repair of Shunt Lesions. Medicina (Kaunas). 2020 Jun 16;56(6):297. doi: 10.3390/medicina56060297. PMID: 32560260; PMCID: PMC7353861.
4. Guo ZK, Chen PG, Li YX, Jiao H, Kong XH, Bai S, Li XF, Liu AJ, Wang GL. Independent prognostic value of lipocalin-2 in congenital heart disease-associated pulmonary artery hypertension. World J Cardiol. 2024 Dec 26;16(12):720-730. doi: 10.4330/wjc.v16.i12.720. PMID: 39734824; PMCID: PMC11669969.
5. Zhu N, Welch CL, Wang J, Allen PM, Gonzaga-Jauregui C, Ma L, King AK, Krishnan U, Rosenzweig EB, Ivy DD, Austin ED, Hamid R, Pauciulo MW, Lutz KA, Nichols WC, Reid JG, Overton JD, Baras A, Dewey FE, Shen Y, Chung WK. Rare variants in SOX17 are associated with pulmonary arterial hypertension with congenital heart disease. Genome Med. 2018 Jul 20;10(1):56. doi: 10.1186/s13073-018-0566-x. PMID: 30029678; PMCID: PMC6054746.
